# Doppler-guided transanal hemorrhoidal dearterilization versus conventional hemorrhoidectomy for treatment of hemorrhoids – early and long-term postoperative results

**DOI:** 10.1186/s12893-019-0469-9

**Published:** 2019-01-10

**Authors:** V. Popov, A. Yonkov, E. Arabadzhieva, E. Zhivkov, S. Bonev, D. Bulanov, V. Tasev, G. Korukov, L. Simonova, N. Kandilarov, A. Taseva, V. Dimitrova

**Affiliations:** 1 Department of General and Hepato-pancreatic Surgery, University Hospital “Alexandrovska”, 1 Georgi Sofiiski Str, 1431 Sofia, Bulgaria; 2 Medical University–Sofia, 15 Acad. I. E. Geshov Bul, 1431 Sofia, Bulgaria

**Keywords:** Hemorrhoids, Conventional hemorrhoidectomy, Transanal hemorrhoidal dearterilization, Postoperative pain, Long-term outcomes

## Abstract

**Background:**

A variety of effective methods for treatment of hemorrhoids has been proposed. In recent years, there has been an increasing number of studies comparing transanal hemorrhoidal dearterilization (THD) and conventional hemorrhoidectomy (CH), but the focus of most studies has been about the early postoperative results. The data about long-term outcomes is still limited. We aimed to compare Doppler-guided THD and CH with regard to early and long-term postoperative results.

**Methods:**

The conducted prospective research included 287 patients who underwent CH (167 cases) or Doppler-guided THD with mycopexy (120 patients) between November 2010 and December 2015. Information on hemorrhoidal stage, demographic data, presenting symptoms, complications, duration of hospital stay, postoperative pain, patients’ satisfaction and follow-up were obtained. Statistical tests were performed by SPSS 19.0.

**Results:**

There was no significant difference between the studied groups according to gender, mean age, preoperative prolapse, pain and pruritus, hemorrhoidal stage and postoperative complications. Preoperative bleeding was more frequent in THD group (*p* = 0,002). The mean visual analog scale (VAS) pain scores in CH and THD groups on days 1, 2 and 7 were 7.01 vs 5.03, 5.07 vs 2.98, 2.39 vs 0,57 (*p* = 0,000). Practically, there was no difference in VAS on day 30 and patients’ satisfaction at the 18th month. Mean hospital stay was 5,13 (CH) and 3,38 days (THD), *p* = 0,000. The postoperative follow-up was between 18 and 78 months (mean 46 ± 16 months). During this stage, 5 patients (2,99%) in CH group required surgery for recurrence. In THD group, 3 patients (2,5%), all with 4th-degree hemorrhoids underwent additional procedures (p 0,802).

**Conclusions:**

Doppler-guided THD seems to be an efficient and safe option for treatment of hemorrhoids, related to lower postoperative pain and excellent, similar long-term outcomes compared to CH. For advanced grades of hemorrhoids, Doppler-guided THD could be a valuable alternative, but there is a need for patients’ selection.

**Trial registration:**

(retrospectively registered) researchregistry3090.

## Background

Hemorrhoidal disease (HD) is the most common disorder of the anal canal [1, 2]. Clinical manifestation includes pain, bleeding, pruritus and prolapse. Throughout the years, a variety of effective methods for treatment of hemorrhoids has been proposed but for grade II, refractory to conservative management, grade III and IV, and in cases of the recurrent disease, the conventional hemorrhoidectomy (CH) remains “the gold standard” [1–3]. However, it is still associated with significant postoperative pain and discomfort, requiring opioids in analgesic management in 20–40% of patients, and complications such as possible sphincter dysfunction, bleeding, infection, anal stenosis, and fecal incontinence [1–3]. In order to reduce them, a Doppler-guided transanal hemorrhoidal dearterilization (THD) has been introduced as an effective alternative since the 90’s [1–4]. There is an increasing number of studies comparing THD and CH, but the focus of most of them is the early postoperative results [5–7]. The data about long-term outcomes and recurrence rates is still limited [5, 6].

The aim of this study was to compare Doppler-guided THD for treatment of hemorrhoids with conventional hemorrhoidectomy in regard to early and long-term postoperative results.

## Methods

The conducted prospective, comparative research included 287 patients who underwent conventional hemorrhoidectomy (CH) or Doppler-guided THD between November 2010 and December 2015. Inclusion criteria were: II, III, IV degree hemorrhoids, both sex, age between 18 and 80 years, ability to understand the procedure, written informed consent. Exclusion criteria were: previous surgery for anal disorders, fecal incontinence, other active anorectal diseases, irritable bowel syndrome. The diagnosis was established by examination and proctoscopy or colonoscopy. The study was non-randomized. After taking an informed consent about the operations, patients underwent the procedures depending on their affordability and will, as well as the surgeon’s personal preference and opinion in each case due to HD status. The duration of the study with a minimum follow-up of 18 months was determined prior to the research. So, the sample size of the study represented all patients operated in our department during this period of time and who met the inclusion criteria. A conventional (Milligan-Morgan’s or Ferguson’s) hemorrhoidectomy was performed in 167 cases (group 1). Doppler-guided THD with mucopexy was carried out in 120 patients (group 2).

The operative technique of the conventional group consisted of a skin incision on the mucocutaneous border, retraction of the pile mass with an artery forceps, dissection and excision of the hemorrhoids to the anorectal junction using an electrosurgical scalpel, Ligasure® or Laser CO2. The base of pedicle was transfixed with an absorbable suture. In Ferguson’s modification, the wound in the mucosa and skin was closed with absorbable sutures after achieving the hemostasis.

Doppler-guided THD was performed using a specific device, consisting of a proctoscope equipped with a Doppler probe and a light source. The technique included a selective ligation using absorbable suture of hemorrhoidal arteries identified by Doppler (at six positions correlating with the odd numbers of the clock in the patient positioned in dorsal lithotomy). If a Doppler signal was detected after the 6th ligation, an additional suture ligation was performed, up to a maximum of eight. The mucopexy was performed with a continuous suture including the redundant and prolapsing mucosa and submucosa.

Postoperative analgesia was achieved by an intravenous usage of Dexketoprofen 50 mg every 8 h, alternating with Metamizole 2 g. In cases of severe, refractory to the previous analgesics pain, especially in the night-time, Pethidine 50 mg has been applied subcutaneously. Information on the stage of hemorrhoidal disease, demographic data, presenting symptoms, complications, duration of hospital stay and follow-up were obtained.

The primary endpoints of the study were the measurements of postoperative pain, early complications (time frame: early postoperative period – 30 days), and long-term outcome (recurrence of HD). Recurrence was defined as internal hemorrhoids seen during the control examinations or on proctoscopy. The levels of pain on the 1st, 2nd, 7th and the 30th postoperative day were graded on a visual analog scale (VAS). The secondary outcome measures were the duration of hospital stay and patients’ satisfaction. Patients’ satisfaction was estimated by a 4-point scale at the 1st and 18th postoperative month. Follow-up was at the 1, 6, 12 and 18 months after surgery, and then annually.

This study received approval of the University Hospital “Alexandrovska” surgical and ethical committee ref.N 40/25.10.2010.

Descriptive statistical methods were used to characterize each variable. Comparison of both groups according to the scores from the VAS on the 1st, 2nd, 7th and the 30th postoperative day was performed by t-test. The threshold for statistical significance was set to *P* < 0.05. Statistical tests were performed by SPSS 19.0 (SPSS Inc., Chicago, Illinois).

## Results

A total of 287 patients underwent surgical procedure due to with II-, III- and IV-graded hemorrhoids during the studied period. The mean age of the patients (186 males and 101 females) was 47,4 years and varied between 18 and 79 years. Group 1 included 104 male and 63 female patients with a mean age of 46,88 years (18 to 79 years) treated with CH. Group 2 included 82 male and 38 female patients with a mean age of 47,77 years (26 to 74 years) who underwent Doppler-guided THD. The clinical characteristics of both studied groups are shown in Table 1.

**Table 1:** Clinical characteristics of both groups of patients treated with CH and Doppler-guided THD (preoperative data)

Clinical characteristics of the studied patients	Group 1 (CH)	Group 2 (Doppler-guided THD)	p
Number of the patients	167	120	
Male/ Female	104/ 63	82/ 38	0.926
Mean age	46.88	47.77	0.404
Pain	134 (80.24%)	85 (70.83%)	0.111
Bleeding	121 (72.46%)	105 (87.5%)	0.002
Pruritus	41 (24.55%)	28 (23.33%)	0.812
Prolapse of anal mucosa	18 (10.78%)	19 (15.83%)	0.208
Grade of the hemorrhoids			0.58
II grade	69	57	
III grade	89	56	
IV grade	9	7	

Constipation was established in 34 (20,36%) of the patients in Group 1 and 24 (20%) in Group 2. (p 0,940) Although it is not a symptom of hemorrhoids, but is an associated factor, since hemorrhoidal symptoms are aggravated by defecation, this leads to hesitance of patients to pass stool and causes constipation. Other reasons for constipation were excluded by colonoscopy in the studied patients.

The postoperative period was uneventful for the most patients. Postoperative complications were observed in 10 cases (5,99%) in Group 1 and in 7 patients (5,83%) in Group 2. (Table 2) Difference in the postoperative morbidity in the groups is not statistically significant (*p* = 0,956).

**Table 2:** Observed early complications (until 30th postoperative day) in THD and CH groups

Type of observed complication	Group 1 (CH)	Group 2 (Doppler-guided THD)	p
Protracted pain and discomfort	4 (2,4%)	3 (2,5%)	0,955
Urine retention	1 (0,6%)	0	0,396
Severe headache on the 2nd postop.day *(probably due to early lift-up of the patient after spinal anesthesia)*	1 (0,6%)	0	0,396
Transitory incontinence	1 (0,6%)	0	0,396
Anal fissure, presenting with pain and mild bleeding	3 (1,8%)	0	0,140
Anal fissure and prolapse of the anal mucosa	0	1 (0,83%)	0,237
Significant recurrent anal bleeding	0	1 (0,83%)	0,237
Weakness in the lower extremities, prolonged pain, and tenesmus	0	1 (0,83%)	0,237
Inflammatory reaction around the sutures, probably with an allergic genesis	0	1 (0,83%)	0,237
Total	10 (5, 99%)	7 (5,83%)	0.956

The mortality rate in both studied groups was 0%. The mean hospital stay for Group 1 and Group 2 was 5,13 days and 3,38 days, respectively (*p* = 0,000)

In the first group, only two of the patients with anal fissures underwent second procedure due to this complication. The other morbidities during the early postoperative period were successfully managed conservatively.

In one case from Group 2, we observed in the postoperative period, an occurrence of an anal fissure and prolapse of anal mucosa (6 mm in size), which had to be treated surgically later. Another patient developed an inflammatory reaction around the sutures, probably with allergic genesis, but his complaints of prolonged pain, tenesmus, and mild bleeding were successfully managed with steroids and metronidazole suppositories. Significant recurrent bleeding was observed in one case on the 20th postoperative day. The patient preoperatively had suffered from IV grade of HD presenting with prolapse and severe blood loss. During the surgery, an abnormality of the blood vessels in the distal rectum was identified. Due to recurrent bleeding, the patient was re-admitted and Milligan-Morgan hemorrhoidectomy was performed. After a new relapse of the symptoms, the patient underwent an excisional procedure again. Later, the same patient was diagnosed with Addison’s disease and coagulopathy. During the follow-up for almost 6 years, there has been no recurrence of HD.

The median VAS scores for pain in the hemorrhoidectomy and Doppler-guided dearterialization plus mucopexy groups on days 1, 2, 7 and 30 are presented in Table 3. As it is seen, the rates of early postoperative pain are significantly lower in THD group. In the end of the 1st month after the operation, practically there is no difference between rates of pain in both groups.

**Table 3:** Mean VAS scores for pain in THD group and CH group

Variable	Group 1 (CH)	Group 2 (Doppler-guided THD)	P
VAS 1st postop.day	7.01 (Std.deviation 0.93)	5.03 (Std.deviation 1.3)	0,000
VAS 2nd postop.day	5.07 (Std.deviation 0.93)	2.98 (Std.deviation 1.14)	0,000
VAS 7th postop.day	2.39 (Std.deviation 0.98)	0.57 (Std.deviation 1.03)	0,000
VAS 30th postop.day	0	0	> 0,05

Patient satisfaction at the 1st and 18th postoperative months, with the use of a 4-point scale, was 3 vs 4 and 4 vs 4 (*p* > 0.05). The mean postoperative follow-up period was 46 ± 16 months (median 45 months, range 18–78 months). With regard to each studied group, the mean follow-up of the patients with CH and THD was 50 ± 17 months (median 51 months, range 18–78 months) and 41 ± 15 months (median 40 months, range 18–78 months), respectively. At the end of the study period, the follow-up was 89,22% (149 patients) in the CH and 90,83% (109 patients) in the THD group but this difference between them is not significant (p 0,655).

During the follow-up, recurrence of the HD was observed in 26 patients (9,06%) – 15 (8,98%) in CH group and 11 (9,17%) in THD group (p 0,957). 5 patients (2,99%) in the CH group required additional surgery due to a recurrent prolapse (2 cases) or frequent bleeding (3 patients). In the THD group a total of 3 patients (2,5%), all with 4th degree HD underwent an additional surgical procedure due to symptom relapse (2 cases with prolapse and 1 with bleeding). We did not establish a significant difference in the re-operation rate (p 0,802). Mild bleeding was observed in 4 patients (2,4%) in CH group and 3 (2,5%) in the THD group (*p* > 0,05). All were treated conservatively. The recurrence in the other cases (6 in CH group and 5 in THD group) was established on the control proctoscopy revealing I-II stage of HD, but the patients were asymptomatic. None of the patients included in the study developed fecal incontinence or anal stenosis.

## Discussion

Since Morrinaga et al. described a novel method of THD in 1995, it has gained popularity as a preferred alternative in the treatment of HD [8]. The improvement of the surgical technique and adding the mucopexy (introduced by Dal Monte et al. in 2007) enable THD to become an option for the management of advanced grades of hemorrhoids, too [9]. First systematic review, published by Giordano et al. in 2009 analyzed 17 articles and showed overall rate of recurrent prolapse of 9% (0 to 37% in different studies), anal bleeding in 0 to 21% of cases and postoperative pain in 18% [10]. However, it is important to note that the mucopexy is not carried out in all cases included in these reports although the highest rate of relapse is observed in patients with IV-grade HD [4]. In the recent years, several studies have been published and reported improved results from this technique [2, 11–15]. Giordano et al. reported postoperative pain rate of 70% in the first postoperative day, tenesmus in 10%, but a recurrence of prolapse of only 3% after a follow-up of almost 3 years [12]. Ratto et al. observed tenesmus in 11,4% of cases and some degree of residual prolapse in 28.6%, but it was significant only in 5.7% [13]. Faucheron et al. reported postoperative pain in only 6% of patients, tenesmus in 1% and recurrence of prolapse in 9% after 34-month follow-up [14].

The real advantages of THD could be estimated in comparison to other methods, proved as effective. Bursics et al. performed a randomized trial (THD vs CH) and also showed similar results after 12 months of follow-up. THD group had an earlier return to normal activities (*p* < 0.0005) and less postoperative pain (*p* < 0.005) [3, 16]. Elmér et al. [7] conducted a non-blinded randomized trial comparing THD and CH. Results showed lower early postoperative pain in THD group, but although the grade of hemorrhoids was significantly reduced after 1 year for both methods, there was a trend to more patients with remaining grade II hemorrhoids in THD group (*p* = 0.06). The recent studies of Trenti et al., 2017 [5] and De Nardi et al., 2014 [6] with relatively long follow-up (about 24 months), showed similar postoperative pain and morbidity, and a similar long-term cure rate of THD and CH. Meta-analysis of Xu et al. (2016) including 4 randomized trials, reported that there are no statistically significant differences in total complications, recurrence and reoperation rates. But it is also noted that patients returned to normal activities faster after THD than after CH [17].

The goal of our study was to compare both methods. However, we have to accept the presence of some heterogeneity in CH group in our research. All patients in this group underwent an excisional hemorrhoidectomy performed by several devices but the basis of the method is the same. The main difference with THD is the lack of tissue excision during it. Furthermore, several studies found no significant difference in postoperative complications and long-term outcome between non-laser and laser/ cautery device and Ligasure hemorrhoidectomy [18, 19]. So, we believe that these minor variations in the technique will not reduce the value of the study. We proved the lower postoperative pain levels in THD group, but in the end of the 1st month practically there is no difference between studied groups. In our opinion, this resulted in the significantly decreased duration of hospital stay for THD group, which was the secondary endpoint of the study. The longest hospital stay in CH group could be explained with the occurrence of postoperative morbidity or the surgeon’s decision for the need of additional observation of the patient in order to avoid development of complications in home conditions in cases with preoperative high-grade HD. The prolonged mean hospital stay, observed for both groups in comparison to other reports, is due to the health insurance system in our country and its requirements for a minimum hospital stay for treatment of the disease. Another reason was a substantial delay from the time of admission to the surgery in some cases.

Regarding the long-term outcomes, we have extended the period of the follow-up (range 18–78 months, mean 46 months) in order to be more precise about them, especially taking into account the achieved 90% follow-up. This makes the research one of the longest studies comparing THD and CH in the literature. The analysis of our study showed that the rates of patients’ satisfaction, HD recurrence, and re-operation rate were similar. The most frequent reason for the re-operation was the prolapse in THD group (2 of 3 patients) and the recurrent bleeding in CH group (3 of 5 cases). Most of the complications and recurrences in THD group and all of these requiring re-operation were observed in patients with IV grade HD. So, we believe that THD might be used in this stage of hemorrhoids, but there is a need for patients’ selection for achieving better results. We also cannot underestimate the THD advantage of preserving the anatomy and physiology of the anal canal, because there is no risk of sphincter damage. The reported rates of incontinence are extremely rare, as it was 0% in our series. However, we have to mention that our study had some limitation – there was no randomization, a limited number of patients with a different grade of hemorrhoids were included and validated questionnaires were not used.

With regards to the value of Doppler guidance in THD procedure, there are controversies in the literature. [3, 4] Theoretically, ligation of all six arteries (on 1, 3, 5, 7, 9 and 11 o’clock in the lithotomy position) could be performed without the expensive Doppler instruments and the same results to be achieved [3, 4, 20]. On the other hand, it has been demonstrated that one-third of the population has at least one artery in an even-numbered clock position, and for this reason, Doppler-guided localization is important in correctly locating the arteries [3, 4, 21]. All of THD procedures in our series have been performed with Doppler assistance, so we cannot share experience without it. But we believe that future large, high-quality, multicenter trials with long-term outcomes are needed to determine whether Doppler guidance in THD is truly necessary or not, as it is concluded in the meta-analysis of Xu et al. [17].

## Conclusions

Doppler-guided THD seems to be efficient and safe option for treatment of hemorrhoidal disease, related to lower postoperative pain and excellent, similar long-term outcomes comparing to the conventional hemorrhoidectomy. For advanced grades of hemorrhoids Doppler-guided THD could be a valuable alternative, but in our opinion, there is a need for patients’ selection.

## Abbreviations

CH: Conventional hemorrhoidectomy
HD: Hemorrhoidal disease
THD: Transanal hemorrhoidal dearterilization
VAS: Visual analog scale
